# Adsorption performance of GO-doped activated ATP composites towards tetracycline

**DOI:** 10.1039/d2ra03023c

**Published:** 2022-07-07

**Authors:** Song Xiaosan, Shui Boyang, Wang Yiru, Zhou Jie, Wang Sanfan, Wu Nan

**Affiliations:** Key Laboratory of Yellow River Water Environment in Gansu Province, Lanzhou Jiaotong University No. 88 Anning West Road Lanzhou 730070 China songxs@mail.lzjtu.cn; School of Environment and Municipal Engineering, Lanzhou Jiaotong University No. 88 Anning West Road Lanzhou 730070 China; Engineering Research Center of Comprehensive Utilization of Water Resources in Cold and Drought Areas, Ministry of Education No. 88 Anning West Road Lanzhou 730070 China

## Abstract

Antibiotic-related environmental contamination directly threatens ecosystems and human health. Adsorption is an efficient and simple treatment process for removing antibiotics from water environments. Attapulgite (ATP) is a natural clay mineral extensively researched as a promising adsorbent material in the food industry, pharmaceutical sanitation, and organic wastewater treatment. Graphene oxide (GO) is widely employed in the treatment of organic wastewater due to its superior physicochemical properties. Here, using high temperature and HCl, ATP was activated (a-ATP), and a GO/a-ATP composite was prepared *via* hydrothermal synthesis. Using an adsorbent dosage of 0.75 g L^−1^, pH = 5, reaction time of 120 min, initial temperature = 35 °C, and initial TC concentration of 50 mg L^−1^, the adsorption capacity of GO/a-ATP for TC was 38.8 mg g^−1^. The pseudo-first-order model (PFO) and pseudo-second-order (PSO) model were fitted to the kinetic data, and yielded an *R*^2^-value of PSO (0.99991) > PFO (0.9389), indicating that the adsorption process is related to chemisorption. Adsorption was also well described by the mixed-order (MO) model (*R*^2^ = 0.9827), demonstrating that two rate-limiting adsorption reaction steps, diffusion and adsorption, occur; the former exerting greater influence. Equilibrium data was fitted to Langmuir, Freundlich, and Temkin isotherm models; the Langmuir model gave the best fit, suggesting the adsorption process is a homogeneous and monolayer adsorption process. Various thermodynamic parameters such as standard Gibbs free energy (Δ*G*^0^) and standard enthalpy (Δ*H*^0^) were also calculated, these results indicate the adsorption reaction is an endothermic process. Our study shows that GO/a-ATP is a promising adsorbent material for use in the adsorption of tetracycline in aquatic environments.

## Abstract 2

Antibiotic-related environmental contamination directly threatens ecosystems and human health. Adsorption is an efficient and simple treatment process for removing antibiotics from water environments.

## Graphical abstract

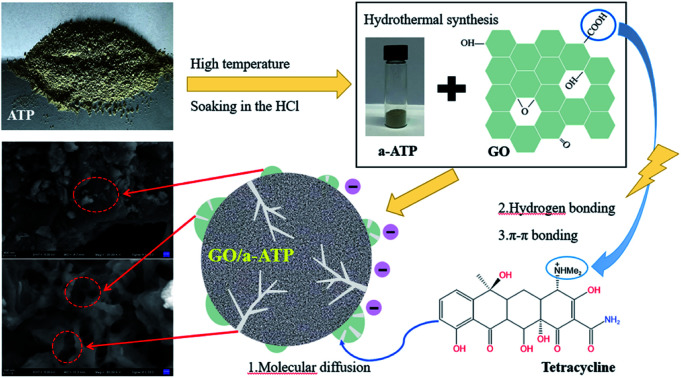

## 1. Introduction

Antibiotics are widely used in livestock agriculture, aquaculture, and medical services as effective preventive and therapeutic medicines for infectious diseases.^1–3^ According to their chemical structure, antibiotics can be classified as tetracyclines, sulphonamides, quinolones, macrolides, β-lactams, and aminoglycosides. Currently, antibiotics' presence can be detected in the effluent of several sewage plants, groundwater, and natural water bodies in China and abroad.^4–7^ Yet, because they do not easily degrade, large amounts of antibiotics in the natural environment can select for highly resistant bacteria or superbugs, which indirectly threaten human health. Moreover, long-term exposure to antibiotics in the aqueous environment could have toxic effects on the cells or genes of organisms especially when their cumulative impact rises.^8^

Tetracycline (TC) is commonly used in pharmaceutical healthcare and industrial farming due to its potent antibacterial effects.^9^ To remove tetracycline and other potentially harmful antibiotics from aqueous environments, researchers have investigated many effective techniques, namely electrochemical,^10,11^ bio-enzymatic,^12^ photocatalytic,^13^ membrane filtration,^14,15^ and adsorption methods.^12,16,17^ Adsorption is a promising strategy for antibiotic removal in water environment because of its low cost, low energy consumption, ease to operate, and high efficiency. Therefore, developing new forms of adsorptive materials that are both cheap and effective is imperative.^18,19^

Attapulgite (ATP) is a naturally occurring magnesium–aluminum silicate clay mineral whose formula is Si_8_(Mg, Al, Fe)_5_O_20_(OH)_2_(OH_2_)_4_·4H_2_O.^20^ Because of its low cost, significant hydrophilicity, and capacity to be further functionalized, ATP is considered an attractive material for water treatment,^21,22^ having been used to treat heavy metal wastewater, excess phosphorus pollution, as well as antibiotics.^23–26^ The adsorption capacity of natural ATP can be improved by high temperature, acid leaching, and organic modification, which will augment its application potential.^27–29^

Graphene oxide (GO), the oxidation product of graphene, exhibits enhanced chemical activity and hydrophilicity.^30^ Under the oxidative stripping effect of concentrated sulfuric acid and potassium permanganate, the original carbon structure is broken by a series of oxygen functional groups, which provides abundant active sites in the basal plane and edge positions. GO and its derivatives have garnered much attention in the field of water treatment.^31,32^ For example, using GO improves the dispersion stability of attapulgite in polyvinyl alcohol (PVA); the PVA/GO-ATT composites were produced by a water blending method, and their mechanical characteristics and thermal stability were found successfully enhanced.^33^ Moreover, doping carbon adsorbent with natural adsorbent materials, such as attapulgite, bentonite, and diatomaceous earth, can help to control costs^34,35^ while also improving the polymerization phenomenon to some extent.^25^

**Fig. 1:**
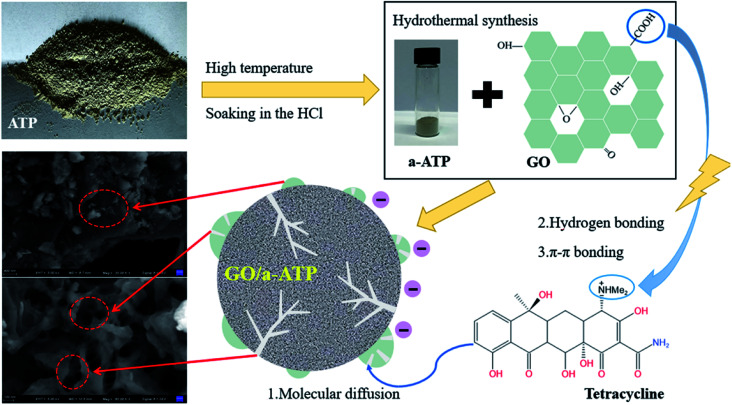
GO/a-ATP preparation and adsorption process on TC.

In this study, samples of an activated a-ATP and GO loaded composite, GO/a-ATP, were successfully prepared by hydrothermal synthesis. The effects of doping ratio, adsorbent dosage, pH, reaction time, temperature, and initial TC concentration upon the samples' adsorption performance for TC were investigated and their adsorption mechanisms analyzed. Our findings provide a new theoretical basis for the treatment of antibiotic-contaminated wastewater (Fig. 1).^36^

## 2. Experimental

### 2.1 Preparation of materials

#### 2.1.1 Preparation of activated ATP (a-ATP)

The natural ATP was calcined in a muffle furnace at 400 °C for 3 h, then mixed with 0.5 mol L^−1^ hydrochloric acid solution and stirred for 1 h. Sonicated for 30 min, and soaked in saturated sodium chloride solution for 30 min, centrifuged and collected the precipitate, rinsed with deionized water, and dried in an oven to obtain activated attapulgite (a-ATP).

#### 2.1.2 Preparation of GO/a-ATP

Graphene oxide (GO) was prepared by the modified hummers method,^37^ where the prepared GO was ultrasonicated for 30 min in deionized water. The activated ATP was dispersed in deionized water, added with sodium hexametaphosphate dispersion stabilizer, and then ultrasonicated at 120 W for 1 h to configure a modified ATP suspension.

The graphene oxide dispersion and the activated ATP suspension were sonicated for 1 h, and then mixed at different mass ratios and sonicated at 80 W. The mixture was stirred at 45 °C, 120 r min^−1^ for 4 h, centrifuged at 4000 r min^−1^, dried at 80 °C, and ground to obtain GO/a-ATP composite adsorbent material.

### 2.2 Chemicals and reagents

**Fig. 2:**
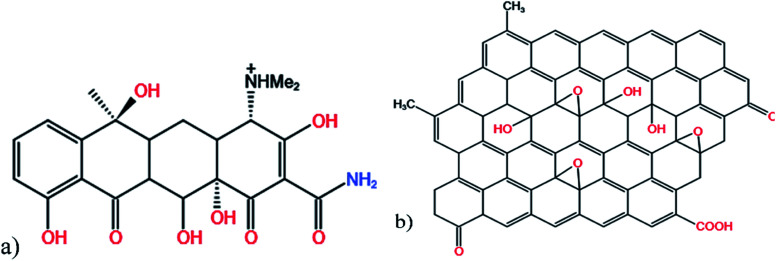
Molecular structure of tetracycline (a) and graphene oxide (b).

Attapulgite was purchased from Baiyin, Gansu; tetracycline was supplied by Maclean Biotechnology Co. Ltd (Shanghai, China); sodium hexametaphosphate, graphite powder, H_2_SO_4_(98% wt), H_3_PO_4_, KMnO_4_, H_2_O_2_, HCl(37% wt), NaCl, NaOH, and anhydrous ethanol were all analytically pure and obtained from Sinopharm Chemical Reagent Company (Beijing, China), all liquid solutions were prepared with deionized water (Fig. 2).

### 2.3 Experimental and analytical methods

#### 2.3.1 Adsorption experiment

0.1 g of tetracycline powder was weighed and dissolved with 0.1 mol L^−1^ hydrochloric acid, then fixed into a 100 mL volumetric flask as a standard solution of tetracycline hydrochloride. We investigated the changes in the adsorption effect under different doping ratio, adsorbent dosing, pH, adsorption time, temperature, and initial concentration conditions. The static adsorption experiment was carried out in a constant temperature oscillator, the reaction vessel was a 250 mL conical flask. The mixture containing tetracycline hydrochloride was added to the flask. The reaction was put into the constant temperature oscillator. The supernatant was filtered and the remaining tetracycline hydrochloride concentration was measured at 375 nm using a 723 N visible light spectrophotometer.

The adsorption capacity of the adsorbent and the removal rate of tetracycline hydrochloride were calculated according to eqn (1) and (2).1
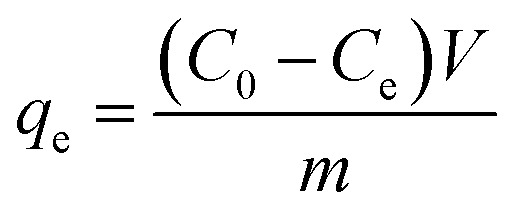
2
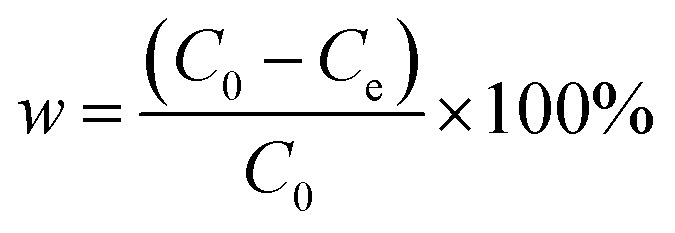
where, *q*_e_ is the adsorption capacity, mg g^−1^; *C*_0_ is the initial tetracycline hydrochloride concentration, mg L^−1^; *C*_e_ is the remaining concentration at the end of the reaction, mg L^−1^; *V* is the vessel volume, mL; *m* is the adsorbent dosage, mg; *w* is the removal rate, %.

#### 2.3.2 Adsorption kinetics

The effect of adsorption time on the final adsorption effect was investigated, and the relationship between residual adsorbate in solution and time was established within 300 min. The experimental data were fitted using the pseudo-first-order (PFO) adsorption kinetic model, the pseudo-second-order (PSO) adsorption kinetic model, and the Weber & Mores internal diffusion model.

For porous adsorption materials, the actual adsorption process is lack homogeneity, so the linear calculation method for PFO and PSO may weaken the non-linear variations present in practice, leading to the appearance of certain deviations,^38^ therefore, the mixed-order model (MO model)^39^ was also used in the study for comparison.^40,41^ The model equations used in the calculations are given in eqn (3)–(6).

3 ln(*q*_e_ − *q*_*t*_) = ln *q*_e_ − *k*_1_*t*

4
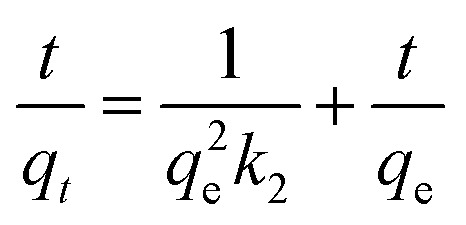

5 *q*_*t*_ = *k*_d_*t*^1/2^ + *C*

6
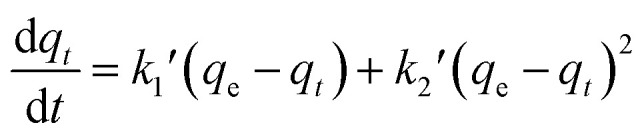
where, *t* is the adsorption time, min; *q*_e_ (mg g^−1^) and *q*_*t*_ (mg g^−1^) are the equilibrium adsorption capacity and the adsorption capacity at the reaction time *t*, respectively; *k*_1_, *k*_2_, and *k*_d_ are the proposed primary kinetic coefficients, the proposed secondary kinetic coefficients, and the particle diffusion coefficients, respectively; *C* is the boundary constant; *k*_1_′(min^−1^) and *k*_2_′(g mg g^−1^ min^−1^) are the primary and secondary kinetic coefficients of the MO model.

#### 2.3.3 Adsorption isotherm

The data were fitted using the Langmuir curve, Freundlich curve, and Temkin curve models. The Temkin isotherm model assumes that the binding energy of the adsorption process is uniformly distributed and the heat of adsorption decreases linearly with increasing molecular coverage of the adsorbent. As Temkin model considers the influence of indirect adsorption on the adsorption behavior and is commonly used to describe the adsorption behavior under non-ideal monolayer adsorption systems.^42,43^ The basic equations of the model are given in eqn (7)–(9).7
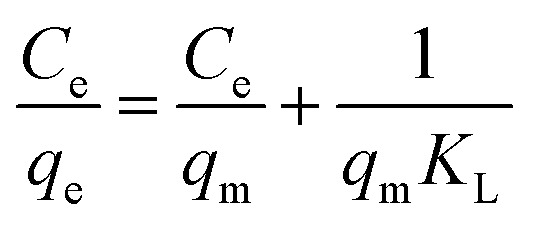
8
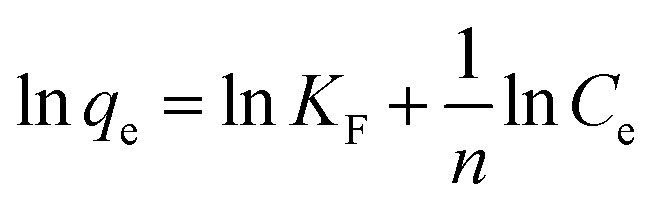

9 *q*_e_ = *B* ln *K*_T_ + *B* ln *C*_e_

where, *C*_e_ is the equilibrium concentration, mg L^−1^; *q*_e_ is the equilibrium adsorption capacity, mg L^−1^; *q*_m_ is the maximum adsorption capacity, mg g^−1^; *K*_L_ is the Langmuir constant, L mg^−1^; *K*_F_ is the Freundlich constant, mg g^−1^; *K*_T_ is the Temkin constant related to the binding energy, L mg^−1^; the constant *B* = *RT*/*b*, kJ mol^−1^, where *b* is the empirical constant, *R* = 8.314 J mol^−1^ K^−1^; *T* is the system temperature, K.

The adsorption process can be further analyzed by the dimensionless parameter *R*_L_. When *R*_L_ = 0, the adsorption process is irreversible, when *R*_L_ > 1 is unfavorable, and when *R*_L_ = 0–1, it indicates that the occurrence of adsorption is favorable, the basic formula is as follows.10
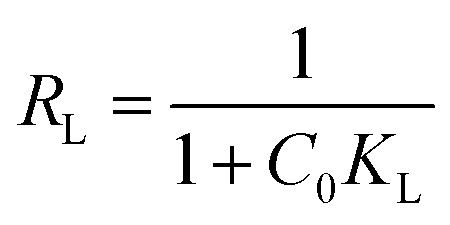
where, *C*_0_ is the initial concentration of the solution, mg L^−1^.

#### 2.3.4 Adsorption thermodynamics

The thermodynamic parameters of the adsorption process are determined to describe the adsorption process. Gibbs energy change (Δ*G*^0^) represents the degree of spontaneity in the adsorption process, with a bigger negative value indicating that the adsorption process is more advantageous to adsorption. The thermodynamic energy state of the reaction can be determined by enthalpy change (Δ*H*^0^), which reveals whether the adsorption process is endothermic or exothermic. Entropy change (Δ*S*^0^) reflects the disorder or degree of disorder at the solid-solution interface during adsorption. Entropy and enthalpy changes are obtained through Van't Hoff equation as follows.11
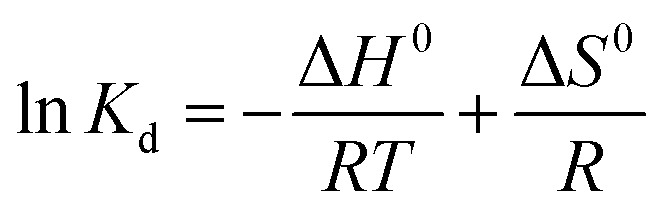
where, *K*_d_ is the dispersion coefficient, L g^−1^; Δ*H*^0^ is the enthalpy change, KJ mol^−1^; *R* is the gas constant, *R* = 8.31 × 10^−3^ KJ mol^−1^ K^−1^; *T* is the absolute temperature, K; Δ*S*^0^ is the entropy change, KJ mol^−1^.

The Gibbs free energy can be calculated by the eqn (12) and (13):

12 Δ*G*^0^ = −*RT* ln *K*_d_

13
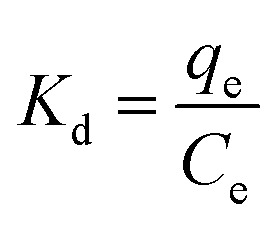
where, Δ*G*^0^ is the Gibbs free energy, KJ mol^−1^.

### 2.4 Characterization of adsorbents

GeminiSEM 500 (ZEISS, Germany) was used to examined the surface morphology of the synthesized samples. X-ray difraction (XRD) patterns were recorded on a MiniFlex600 powder diffractometer (Rigaku, Japan) from 5° to 80° (in 2*θ*) with the scanning rate of 5° min^−1^. Fourier transform infrared (FT-IR) spectra of each sample were recorded on VERTEX 70 FT-IR spectrometer (Bruker, Germany) with the KBr pellet technique. Zeta potential was analyzed by Zetasizer Nano S90 (Malvern, British).

Raman spectra was analyzed by DXR™ 3(Thermo Fisher, America).

## 3. Results and discussion

### 3.1 Characterization

#### 3.1.1 SEM

**Fig. 3:**
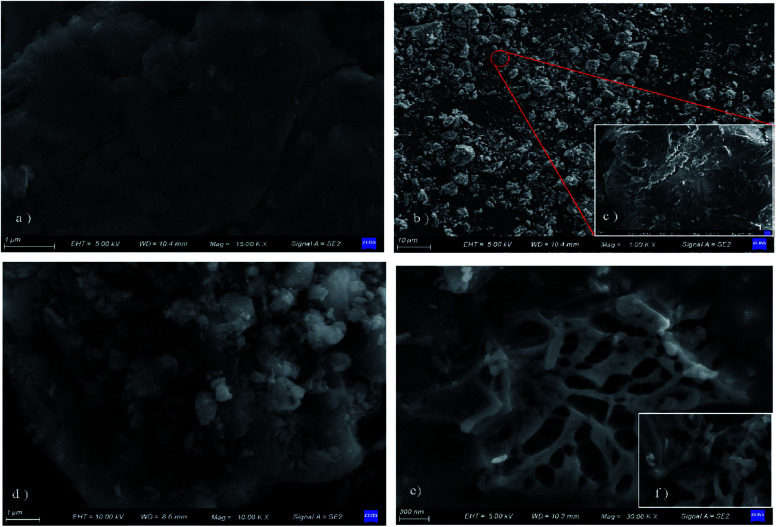
SEM of GO (a), GO/a-ATP (b–f).

Fig. 3 shows the surface morphology and microstructural conditions of GO and GO/a-ATP. Evidently, the GO produced by the modified Hummers' method has a smooth surface with a stacked lamellar structure (Fig. 3a). By contrast, the prepared GO/a-ATP has much larger particle sizes and a coarse surface after grinding, likely due to the doping load of a-ATP, with most of its particles being 1–10 μm in size (Fig. 3b–d). Large pore structures with sizes of *ca.* 100–300 nm were observed under magnification (Fig. 3e and f). The high-temperature calcination and acid modification of ATP generated that pore structure, thereby facilitating the diffusive adsorption of TC molecules onto GO/a-ATP.

#### 3.1.2 XRD

**Fig. 4:**
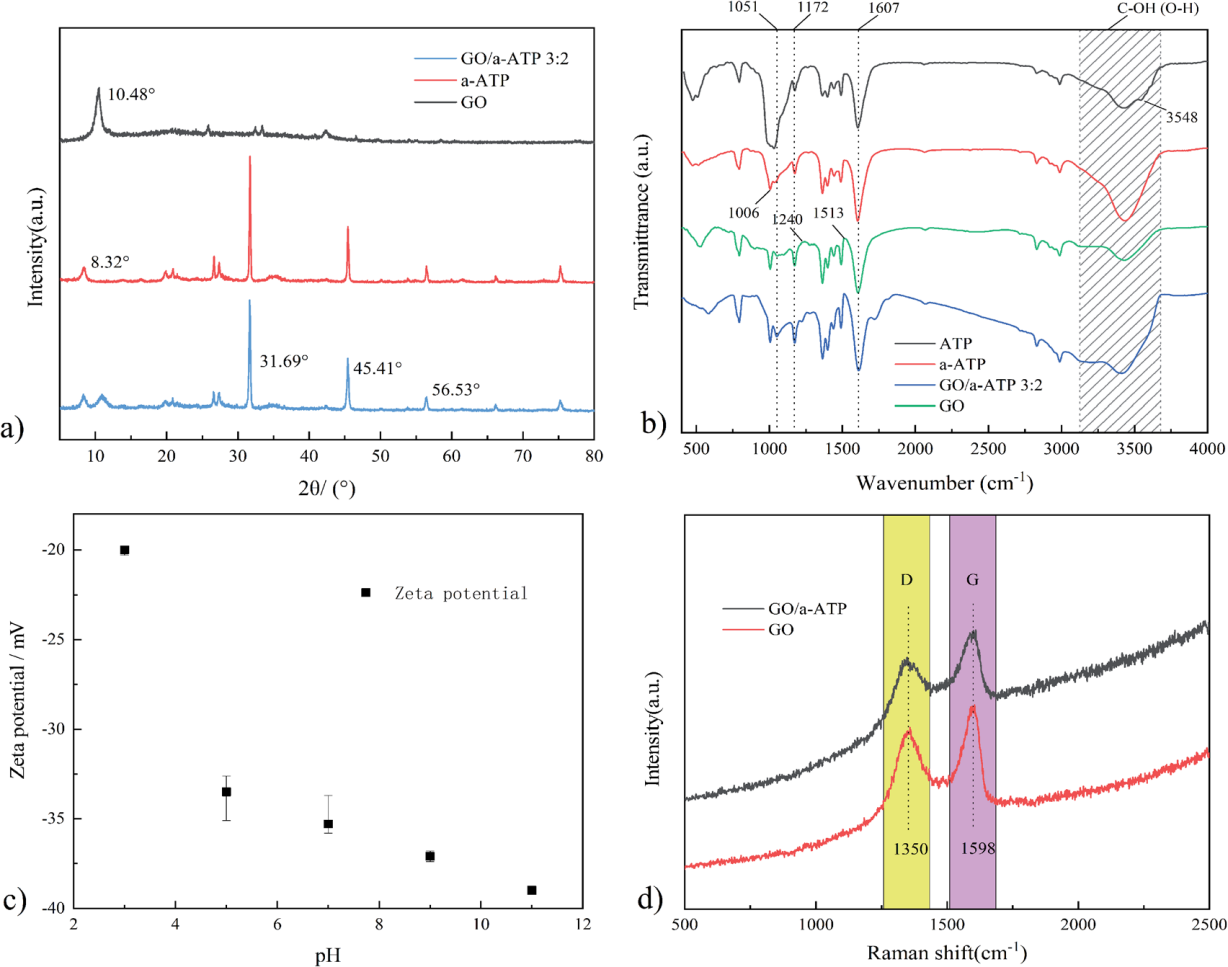
XRD (a), FTIR (b), zeta potential (c) and Raman spectra (d) of GO/a-ATP.

In this work, XRD was used to examine the structural changes in the crystalline material of each sample (Fig. 4a). Due to the irregular stacking of π–π interactions and hydrogen bonding, the GO shows the (001) crystallographic plane diffraction peak at 2*θ* = 10.48°. According to Bragg's equation, the GO layer spacing is 0.84 nm.

The diffraction peak of a-ATP at 2*θ* = 8.32° corresponds to the (110) crystallographic plane.^44^ The structural characteristic peaks appear at 2*θ* = 31.69° and 45.41° and are preserved in the GO/a-ATP composite, for which the smaller area of the diffraction peak indicates there is less a-ATP. This implies the successful composite of GO and a-ATP.

#### 3.1.3 FTIR

The FT-IR spectra of ATP, a-ATP, GO, and GO/a-ATP composites were compared to verify the relevant chemical functional groups. As shown in Fig. 4b, there is a broad peak centered at 3300 cm^−1^, which corresponds to the stretching vibration peaks of C–OH (O–H) and adsorbed H_2_O, and the acid-modified ATP shows stronger absorption near 3300 cm^−1^, which is also consistent with the literature.^45^

Pure ATP had absorption peaks at 1172, 1607, and 1006 cm^−1^, corresponding to the non-freezable water, Si–OH, and Al(Mg)–OH groups in the ATP framework. These characteristic groups of pure ATP were largely retained on a-ATP after it underwent high temperature and acid leaching. Compared with pure ATP, the disappearance of the asymmetric stretching vibration peak at 3548 cm^−1^ of a-ATP was attributed to the ion exchange that occurred in ATP; the introduction of small radius cations (H^+^), these increasing both the pore area and specific surface area.^46^

The peaks of GO located near 1513, 1607, 1051, 1240, and 1172 cm^−1^ correspond to the absorption peaks of the aromatic structure C=C backbone vibrations and the characteristic peaks of O–H bonding, the stretching vibrational peaks of C=O on carboxyl groups, and the stretching vibrational peaks of C–O and C–O–C, respectively.^44,47^ Peaks near 1607, 1051, 1240, and 1172 cm^−1^ suggest the existence of a substantial number of oxygen-containing functional groups in the GO interlayer and edge.^28^ These various oxygen-containing GO groups would offer active sites for grafting reactions between GO and ATP.

The results by FTIR showed that a-ATP was successfully inserted on the GO nanosheets *via* a grafting modification process with GO oxygen-containing functional groups, and GO/a-ATP retained the characteristic groups of a-ATP and GO.

#### 3.1.4 Zeta potential

In Fig. 4c, we can see that the electronegativity of the GO/a-ATP surface gradually increases as the pH rose, from −20 mV at pH = 3 to −39 mV at pH = 11. Accordingly, given that material's surface is negatively charged, it should be more apt to adsorb positively-charged TCsH_3_^+^ and TCsH_2_^±^ molecules, and the density of negative charges on the surface of GO/a-ATP increases with a rising pH, and the solvation stability of the prepared composites is enhanced.

#### 3.1.5 Raman spectra

Raman analyses were preformed for GO and the GO/a-ATP composite. As Fig. 4d shows, two characteristic peaks arose at 1350 cm^−1^ and 1598 cm^−1^, respectively corresponding to the D-band and G-band of graphene oxide. The D-band is a disordered vibration peak generated by carbon defects, while the G-band is an elastic vibration produced by graphene's sp^2^ structure. The relative intensity ratio of the D-band to the G-band (*I*_D_/*I*_G_) conveys the degree of carbon defects in the material. The *I*_D_/*I*_G_ of GO/a-ATP (0.89) is nearly identical to that of GO (0.88), thus confirming there was no significant effect on the carbon structure of the GO material after doping it with a-ATP.

### 3.2 Adsorption capacity of the different adsorbent

**Fig. 5:**
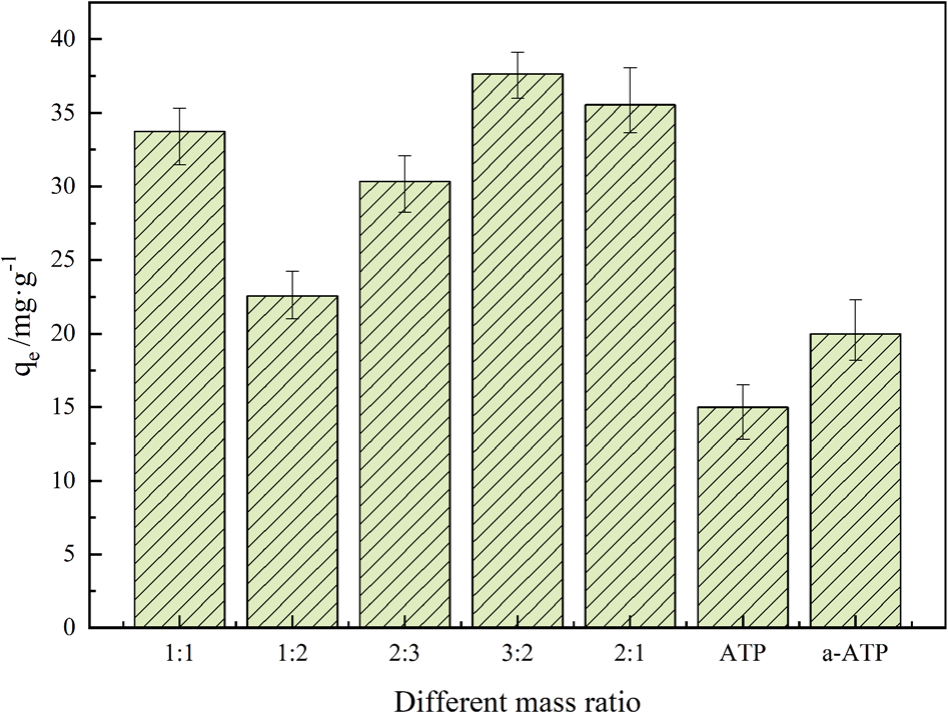
Adsorption capacity of GO/a-ATP at different mass ratio (pH = 3, *t* = 120 min, *T* = 25 °C, TC concentration = 30 mg L^−1^, dosage = 0.5 g L^−1^).

Adsorbents with differing doping ratios of GO and a-ATP were prepared and their adsorption capacities for TC was investigated, which were also shown in Table 1 and Fig. 5. The adsorption capacities of a-ATP (20.13 mg g^−1^) was enhanced significantly compared to natural ATP (14.89 mg g^−1^). This suggests the hydrochloric acid and high temperature modifications effectively removed impurities from natural ATP, for more TC-adsorbing sites.^45,48^

**Table 1:** The adsorption capacity of different materials for TC

Materials	ATP	a-ATP	GO/a-ATP 1 : 1	GO/a-ATP 1 : 2	GO/a-ATP 2 : 3	GO/a-ATP 3 : 2	GO/a-ATP 2 : 1
Adsorption capacity (mg g^−1^)	14.89	20.13	33.76	22.58	30.34	37.64	35.58

The adsorption capacity of a-ATP was increased significantly after doping with graphene oxide, rising further with more of the latter used, reaching a maximum of 37.64 mg g^−1^.

As shown in Fig. 5, the adsorption capacity rises and then diminishes, peaking at a doping ratio of 3 : 2 for GO to a-ATP. This could be explained by a-ATP playing a major role in the adsorption of TC when the GO addition is low; as GO doping ratio increases, the extra functional groups and large pore structure on GO substantially strengthened the adsorption capacity.^49^ However, excessive GO doping can cause material agglomeration, which would reduce the adsorption capacity. In the following studies, the composite adsorbents used were prepared in a 3 : 2 ratio.

### 3.3 Adsorption capacity of the different dosages

**Fig. 6:**
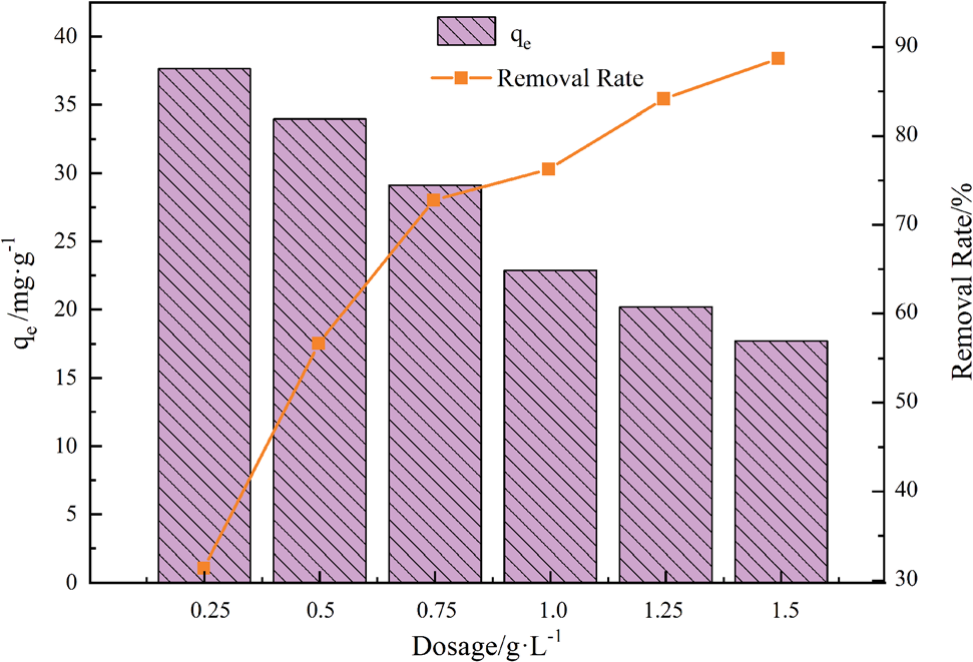
Influence of adsorbent dosage on adsorption for GO/a-ATP (pH = 3, *t* = 120 min, *T* = 25 °C, TC concentration = 30 mg L^−1^).

The adsorbent dosage curves appear in Fig. 6. Evidently, the adsorbent dosage directly modulated the adsorption effect of the composite material on TC. With the increase of the dosage from 0.25 to 1.5 g L^−1^, the adsorption rate showed an increasing trend, but the adsorption capacity showed a decreasing trend. This could be because as the GO/a-ATP dosage increases, there is more of the contact area exposed and available to adsorb TC; hence, its removal efficiency rises as the dosage increases. However, the presence of significant amounts of GO/a-ATP can cause agglomeration, leading to adsorption sites overlapping and a reduction of the adsorbents' effective surface area, thus diminishing the adsorption capacity.^34^

### 3.4 Effect of pH on the adsorption capacity

The adsorption effects of pH levels of 3, 5, 7, 9, and 11 were investigated. The adsorption efficiency of the composites for TC increased and then decreased as they pH rose. When pH is 5, the maximum adsorption capacity and removal rate is 32.28 mg g^−1^ and 78.08%, respectively.

**Fig. 7:**
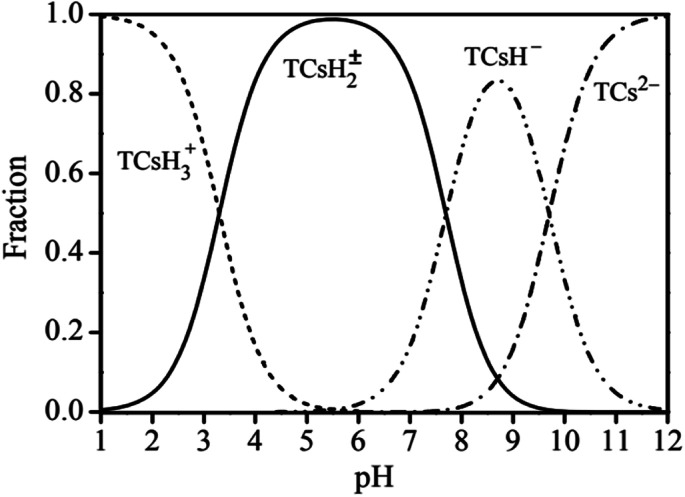
Different forms of TC at different pH.

**Fig. 8:**
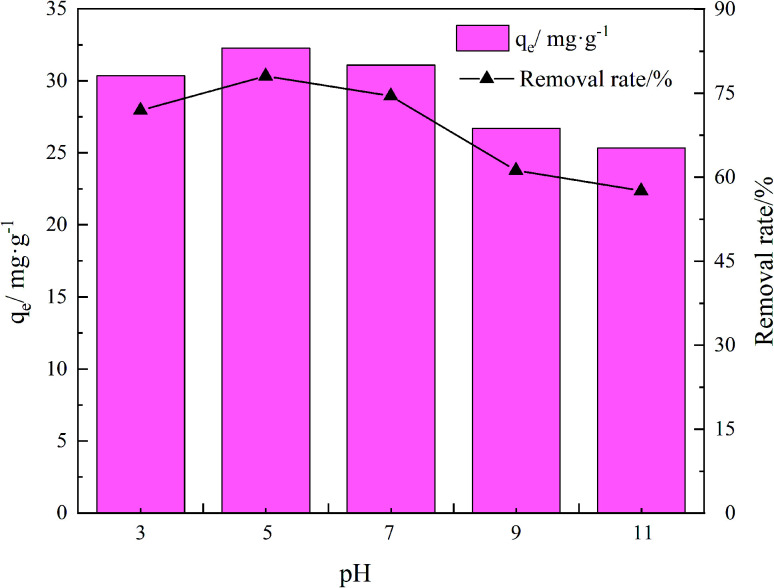
Influence of pH on adsorption for GO/a-ATP (*t* = 120 min, *T* = 25 °C, dosage = 0.75 g L^−1^, TC concentration = 30 mg L^−1^).

Because TC exists in different forms under different pH conditions, the mechanism by which its adsorption occurs also has differences. As shown in Fig. 7 and 8, the amine group in TC can protonate with H^+^ when pH < 3.3 and exist mainly in the form of positively charged TCH_3_^+^, when the molecular polarity is greatly enhanced. Given the negative charge on the surface of GO/a-ATP material, electrostatic adsorption with TCH_3_^+^ can happen, but a large amount of H^+^ will still compete with TCH_3_^+^ for adsorption, leading to fewer active site available.

When the pH is = 3.3–7.7, the system is gradually dominated by the amphiphilic form of TCH_2_^±^. In this range, as the H^+^ concentration is reduced, the competitive adsorption effect gradually decreases, and the adsorption efficiency of GO/a-ATP for TC is improved. When pH > 7.7, the anionic form of TCH^−^ and TC_2_^−^ mainly exist,^19,50^ conferring to the material's surface many negative charges. Accordingly, the rate at which TC is adsorbed decreases due to the repulsive force between the material and TC molecules.

### 3.5 Effect of reaction time

**Fig. 9:**
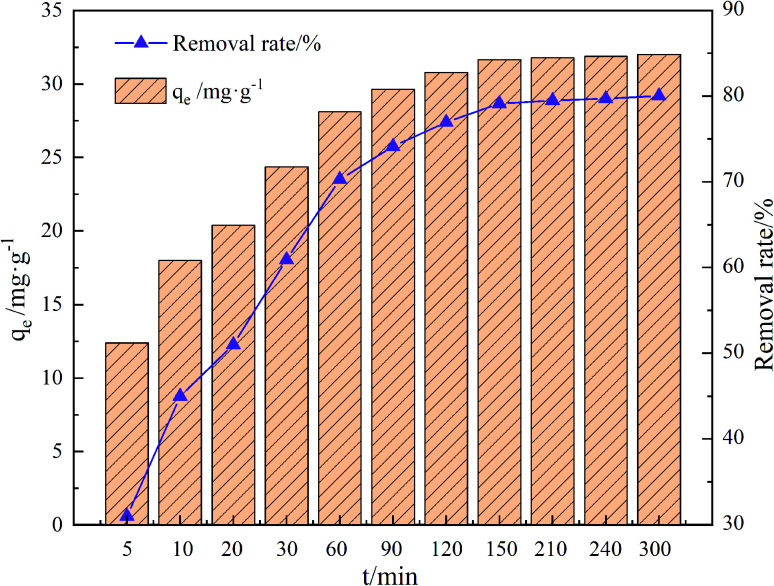
Influence of time on adsorption of GO/a-ATP (pH = 5, *T* = 25 °C, dosage = 0.75 g L^−1^, TC concentration = 30 mg L^−1^).

As shown in Fig. 9, the adsorption of GO/a-ATP on TC increased rapidly over the first 60 min, with the adsorption equilibrium finally reached at 120 min, corresponding to a maximum capacity of 30.78 mg g^−1^. This fast start to the amount adsorbed was most likely driven by electrostatic interaction between the negatively-charged surface and the positively-charged TC ions. In this way, most adsorption sites on the composite adsorbent would have quickly gotten filled, with prolonged contact time needed to inevitably, but gradually, attain equilibrium. At this point, the adsorption capacity increases extremely slowly or stops increasing altogether. The optimum equilibrium reaction time was determined to be 120 min from the following investigation.

**Fig. 10:**
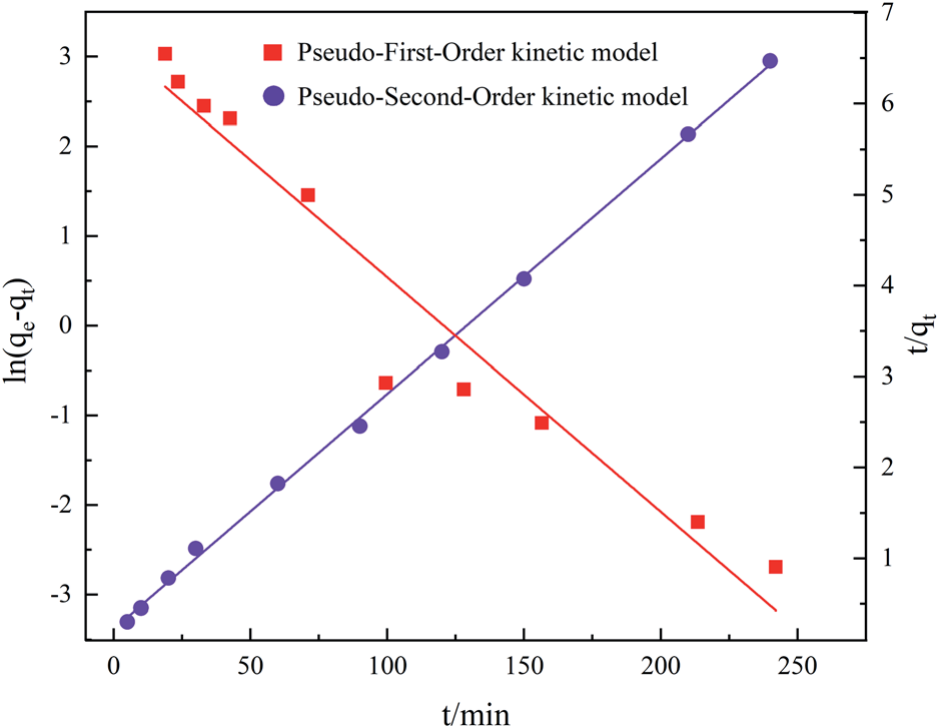
Kinetic model of TC adsorption by GO/a-ATP.

As shown in Fig. 10, more points fell on the pseudo-second-kinetic model's curve. According to Table 2, its coefficient of determination (*R*^2^) was 0.9991 for TC adsorption, this higher than the 0.9389 obtained for the pseudo-first-kinetic model. Hence, the proposed secondary kinetic model best describes the adsorption of TC onto GO/a-ATP composites.

**Table 2:** Kinetic parameters of TC adsorption by GO/a-ATP

Pseudo-first-order kinetic model			Pseudo-second-order kinetic model			
*q*_e_	*k*_1_	*R* ^2^	*h*	*q*_e_	*k*_2_	*R* ^2^
mg g^−1^	min^−1^	—	mg (g^−1^ min^−1^)	mg g^−1^	g (mg^−1^ min^−1^)	—
20.29	2.7135	0.9389	4.4543	38.68	0.00298	0.9991

It can be shown that GO/a-ATP has abundant active sites for binding TC and that their adsorption is the main mechanism and rate-controlling step of the adsorption process. This chemisorption is also related to the electron transfer and interaction between the composite GO/a-ATP and TC.^41,51^ The initial rate of this reaction was determined to be 4.4543 mg g^−1^ min^−1^ according to the formula *h* = *k*^2^*q*_e_^2^.

Because of their simple analytical structure, the pseudo-first-kinetic model and pseudo-second-kinetic model, can only adequately characterize the boundary properties of typical kinetic experiments. The mixed-order (MO) model, which integrates first- and second-order reactions, divides the reaction process into two partial stages, fast and slow adsorption, and some studies find it better suited for conveying the adsorption process.^39,40^

The fitting results of the mixed-order model are shown in Fig. 12 and Table 4. In the former, almost all points fell on the curve, for an *R*^2^ = 0.9827. This is perhaps expected, because diffusion and adsorption processes are known to influence the adsorption process according to the quasi primary and quasi secondary rate constants of the MO model.^39^

**Fig. 11:**
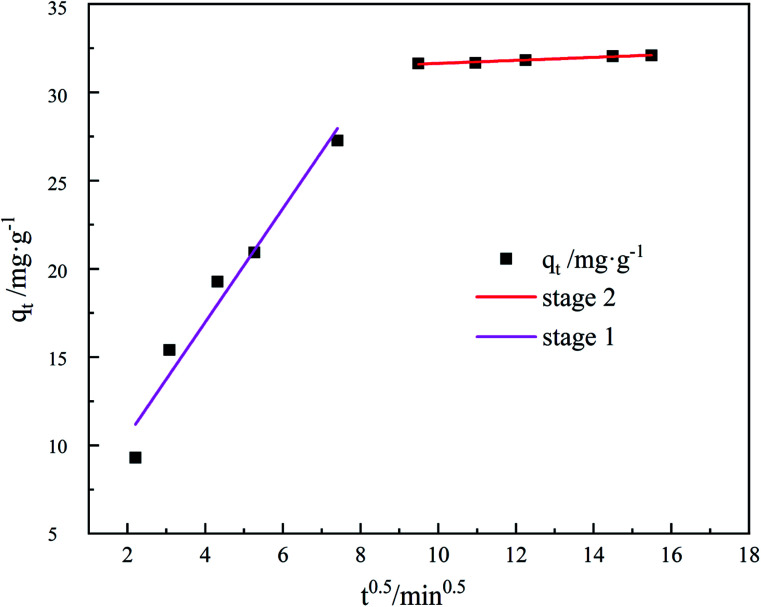
Weber & Mores diffusion model of TC adsorption by GO/a-ATP.

To describe the diffusion of the system in a specific range, the adsorption process was fitted with an internal diffusion model. The results are presented in Fig. 11 and Table 3, evidently, the straight line fitted by *q*_*t*_ to *t*^0.5^ divides into two segments and does not pass through the origin, indicating that the adsorption rate is influenced by more than one element of internal diffusion.

**Fig. 12:**
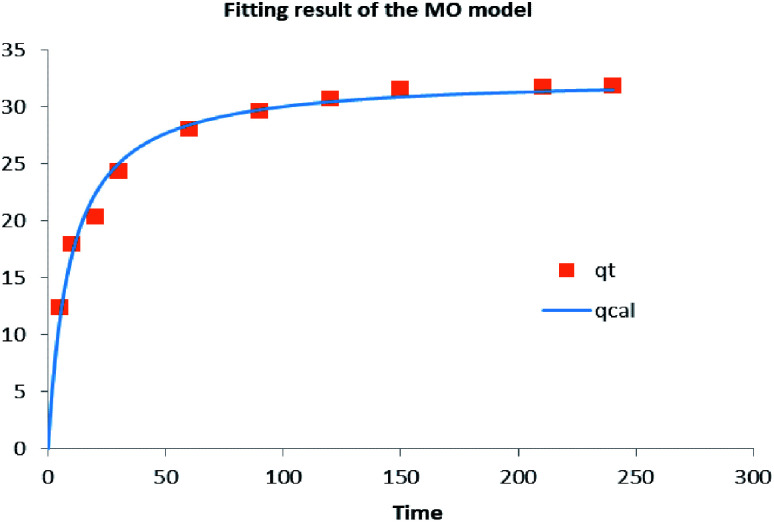
Mixed-order model of TC adsorption by GO/a-ATP.

**Table 3:** Weber & Mores diffusion parameters of TC adsorption by GO/a-ATP

First stage			Second stage		
*k*_d1_	*C*_1_	*R* ^2^	*k*_d2_	*C*_2_	*R* ^2^
mg g^−1^ min^−1/2^	—	—	mg g^−1^ min^−1/2^	—	—
2.7895	11.8899	0.9423	0.08463	35.7936	0.9628

**Table 4:** Mixed-order model parameters of TC adsorption by GO/a-ATP

Mixed-order model					
*K*_1_	*k*_2_	*R* ^2^	SSE	MSE	*χ* ^2^
min^−1^	g mg^−1^ min^−1^	—	—	—	—
0.00639	0.00319	0.9827	7.6371	0.7637	0.3880

The TC undergoes external diffusion in aqueous solution and is adsorbed onto the composite surface of GO/a-ATP in the first stage; in the second stage, the adsorbed TC undergoes internal diffusion between the pore channels or layers of the GO/a-ATP composite. The internal diffusion model's adsorption parameters showed that *k*_d1_ in the first stage was greater than *k*_d2_ in the second, indicating that a high rate of TC adsorbed by GO/a-ATP in the initial period.

### 3.6 Effects of temperature and initial TC concentration

**Fig. 13:**
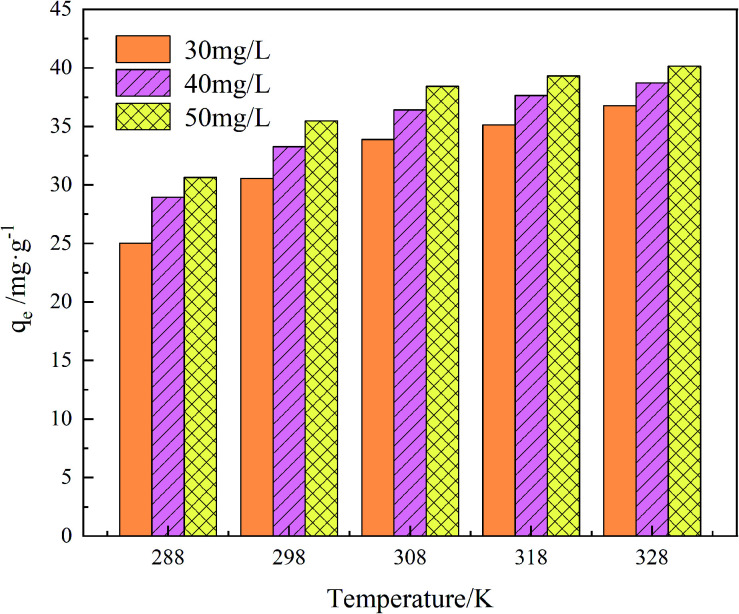
Influence of temperature and initial TC concentration on adsorption of GO/a-ATP (pH = 5, *t* = 120 min, dosage = 0.75 g L^−1^).

The adsorption effect of GO/a-ATP on TC at 15 °C, 25 °C, 35 °C, 45 °C, and 55 °C was examined (Fig. 13). The adsorption capacity increased with higher temperatures and reached 27.94 mg g^−1^ (15 °C), 33.27 mg g^−1^ (25 °C), 35.42 mg g^−1^ (35 °C), 37.65 mg g^−1^ (45 °C), and 38.73 mg g^−1^ (55 °C) at an initial TC concentration of 40 mg g^−1^, respectively.

**Fig. 14:**
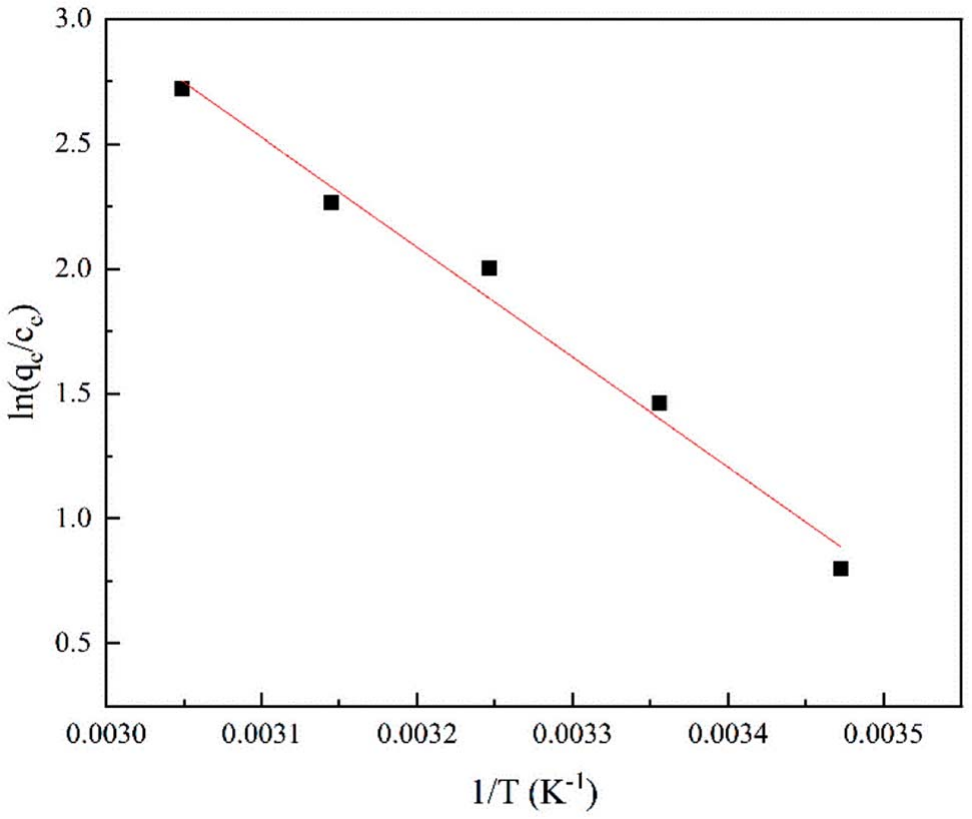
Thermodynamic model of TC adsorption by GO/a-ATP.

When the ambient temperature is constant, the initial TC concentration affects the adsorption mass transfer process and thus the adsorption effect of the adsorbent on TC. As Fig. 13 shows, at 35 °C, the adsorption capacity of GO/a-ATP was 33.89, 36.52, and 38.43 mg g^−1^ for an initial TC concentration of 30, 40, and 50 mg g^−1^. The adsorption capacity is strengthened with more TC present but this improvement wanes, perhaps due to the saturation of adsorption sites of GO/a-ATP (Fig. 14).

Table 5 conveys the results of the thermodynamic parameter calculations. That the enthalpy change of the adsorption reaction was Δ*H*^0^ > 0 indicated that the TC-adsorption process of GO/a-ATP is a heat absorption reaction. Increasing the reaction temperature is thus beneficial to the reaction. The Gibbs energy change of Δ*G*^0^ < 0, and the absolute value of Δ*G*^0^ increased gradually with a rising temperature, which suggests that the adsorption process of TC onto GO/a-ATP could proceed spontaneously, with higher temperature contributing to a stronger adsorption effect.

**Table 5:** Thermodynamic parameters of TC adsorption by GO/a-ATP

Temperature (K)	Δ*G*^0^/(KJ mol^−1^)	Δ*H*^0^/(KJ mol^−1^)	Δ*S*^0^/(J mol K^−1^)	*R* ^2^
288	−1.91923	36.5681	0.1344	0.9809
298	−3.6215			
308	−5.12565			
318	−5.9831			
328	−7.41628			

**Fig. 15:**
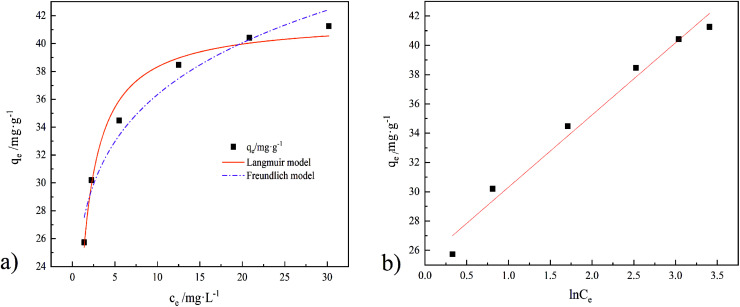
Isotherm models of TC adsorption by GO/a-ATP, ((a), Langmuir and Freundlich model. (b), Temkin model); *T* = 35 °C.

In Fig. 15 are the results of three models (Langmuir, Freundlich, and Temkin) fitted to the adsorption data of GO/a-ATP composites, with their corresponding parameters given in Table 6. Clearly, more points fell exactly on the linear regression of the Langmuir model, whose *R*^2^-value was highest, followed by Temkin model, and lowest for the Freundlich model (Table 6). Hence, the Langmuir model and Temkin model can better describe the process by which TC is adsorbed onto GO/a-ATP.

Isothermal parameters of TC adsorption by GO/a-ATP

**Table:** 

Langmuir model				Freundlich model		
*q*_m_	*K*_L_	*R* ^2^	*R*_L_	*K*_F_	1/*n*	*R* ^2^
mg g^−1^	L mg^−1^	—	—	mg g^−1^	—	—
41.75	1.1166	0.9798	0.0176	26.29	0.1401	0.9520

**Table:** 

Temkin model		
*K*_T_	*B*	*R* ^2^
L mg^−1^	kJ mol^−1^	—
25.3811	4.9317	0.9736

Langmuir model suggests a unimolecular layer adsorption and that surface of the material contains a limited number of uniform adsorption sites. The maximum adsorption capacity calculated using the Langmuir model is 41.75 mg g^−1^, being closer to the experimental value than that predicted by the Freundlich model's. According to the calculation result from the dimensionless parameter *R*_L_, the adsorption of GO/a-ATP on TC is a favorable adsorption process with a strong affinity between this adsorbent and TC.

### 3.7 Recycling performance

In practical applications, the loss of the adsorbent during adsorption puts a lot of pressure on the water environment. Therefore, reusability is a critical factor for an optimal adsorbent. In this experiment, a 0.1 M NaOH solution was used for desorption while sonicating for 60 min. The recovery process of the material consists of several steps: first, adsorption of TC on the surface and pores of the adsorbent material; second, centrifugal separation of GO/a-ATP from the wastewater; third desorption of TC in NaOH; and, finally, filtration and drying to obtain the regenerated GO/a-ATP.

**Fig. 16:**
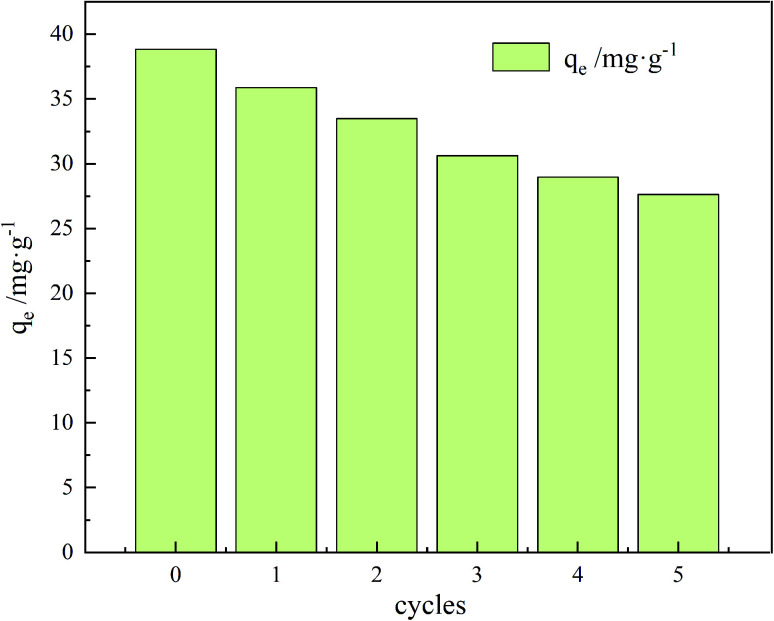
Adsorption–desorption cycle of GO/a-ATP towards TC (pH = 5, *C*_0_ = 50 mg L^−1^, *t* = 120 min, *T* = 35 °C, dosage = 0.75 g L^−1^).

As evinced by Fig. 16, after five adsorption–desorption cycles, the adsorption capacity still reached 71% of its initial magnitude. These results indicate that the GO/a-ATP could be used repeatedly, four rounds of adsorption–desorption, without significantly decreasing its adsorption capacity. This robust retention could be an effect of the hydrogen bond and π–π bond between TC and GO/a-ATP. The gradual decrease in the rate of desorption might be driven by fewer available adsorption active sites or residual TC caused by incomplete desorption.

## 4. Conclusion

In this work, a form of activated ATP (a-ATP) was obtained *via* calcination and acid leaching of pure ATP, significantly enhancing its capability to adsorb TC (tetracycline). We then synthesized GO/a-ATP composites by hydrothermally doping GO with a-ATP, and investigated their TC-adsorption properties.

Multiple effects, adsorbent dosage, pH, reaction time, temperature, and initial TC concentration were examined. The adsorption capacity of GO/a-ATP for TC was greatest in a doping ratio of 3 : 2. When using that ratio, along with a dosage of 0.75 g L^−1^, a pH of 5, reaction time of 120 min at 35 °C, and the initial TC concentration of 50 mg L^−1^, the adsorption capacity of GO/a-ATP was 38.8 mg g^−1^. Moreover, after five adsorption–desorption cycles of regeneration, the GO/a-ATP composite for TC could still maintain at least 71% of its initial adsorption capacity.

The adsorption of TC by GO/a-ATP composites follows the pseudo-second-order model and the Langmuir model, indicating that TC adsorption onto GO/a-ATP was predominantly a chemical and monolayer adsorption process. Under ideal conditions, the adsorption capacity could reach 41.75 mg g^−1^ according to the Langmuir model. The thermodynamic fitting results indicate that the adsorption of TC by GO/a-ATP is a heat absorption process, therefore a higher ambient temperature promotes the reaction. Taken together, our results highlight that the activation of natural ATP can enhance its adsorption capability, and that the prepared GO/a-ATP is a promising material for use as a potential adsorbent to remove TC from wastewater and aquatic environments.

## Author contributions

Conceptualization, Shui Boyang and Song Xiaosan; methodology, Shui Boyang; software, Song Xiaosan; validation, Song Xiaosan, Wang Yiru and Wu nan; formal analysis, Wang Sanfan; investigation, Shui Boyang; resources, Shui Boyang; data curation, Wang Yiru; writing—original draft preparation, Song Xiaosan; writing—review and editing, Shui Boyang; visualization, Song Xiaosan; supervision, Wang Sanfan; project administration, Wang Sanfan; funding acquisition, Wang Sanfan. All authors have read and agreed to the published version of the manuscript.

## Conflicts of interest

No potential conflict of interest was reported by the authors.

## Supplementary Material
